# Supplementary material for: Brain Tissue Oxygenation-Guided Therapy and Outcome in Traumatic Brain Injury: A Single-Center Matched Cohort Study
Source: Brain Sci. 2022 Jul 6;12(7):887. doi: 10.3390/brainsci12070887 (PMC9315682; doi:10.3390/brainsci12070887)

Electronic Supplemental Material - Brain Tissue Oxygenation-Guided Therapy and Outcome in Traumatic Brain Injury: a single-center matched cohort study

Table S1: Logistic regression for the association of $PbtO_2$ monitoring and hospital mortality.

	Univariable analysis<br>OR (95% CI)	Multivariable analysis<br>OR (95% CI)
Age	1.05 (1.02-1.07)	1.06 (1.03-1.10)
GCS on admission	0.93 (0.83-1.03)	0.83 (0.72-0.96)
ICHT	7.23 (2.65-19.71)	9 (2.69-30.9)
PbtO2 monitoring	0.51 (0.22-1.18)	1.33 (0.46-3.87)

GCS: Glasgow coma scale; ICHT: intracranial hypertension; $PbtO_2$: brain tissue oxygenation monitoring

Table S2: Logistic regression for the association of $PbtO_2$ monitoring and unfavorable neurological outcome (GOS 1-3) at 3 months.

	Univariable analysis OR (95% CI)	Multivariable analysis OR (95% CI)
Age	1.04 (1.01-1.06)	1.06 (1.03-1.10)
GCS on admission	0.86 (0.77-0.96)	0.74 (0.63-0.88)
ICHT	4.84 (1.99-11.72)	4.93 (1.65-14.71)
PbtO2 monitoring	0.43 (0.19-1.01)	0.71 (0.25-2.03)

GCS: Glasgow coma scale; ICHT: intracranial hypertension; $PbtO_2$: brain tissue oxygenation monitoring

Table S3: Characteristics of patiens admitted from February 2017 to December 2019. Data are presented as mean (±SD), count (%) or median (25th-75th percentiles).

	ICP-group (N=24)	ICP/$PbtO_2$-group (N=35)	p value
**Age, years**	55 (±21)	45 (±17)	0.02
**Male gender, n (%)**	14 (58)	24 (69)	0.58
**Arterial hypertension, n (%)**	10 (42)	7 (20)	0.09
**Diabetes mellitus, n (%)**	2 (8)	2 (6)	0.99
**Heart disease, n (%)**	1 (4)	2 (6)	0.99
**Previous neurological disease, n (%)**	1 (4)	2 (6)	0.99
**Alcohol use, n (%)**	7 (29)	10 (29)	0.99
**Smoking, n (%)**	6 (25)	5 (14)	0.33
**COPD, n (%)**	0	0	-
**Liver cirrhosis, n (%)**	2 (8)	1 (3)	0.56
**Cancer, n (%)**	1 (4)	1 (3)	0.99
**Chronic kidney disease, n (%)**	1 (4)	0	0.41
**APACHE score**	16 (16-21)	20 (17-23)	0.18
**SOFA score**	9 (4-10)	8 (8-10)	0.60
**GCS on admission**	5 (3-10)	5 (3-7)	0.84
**Marshall score, n (%)**<br>**1**<br>**2**<br>**3**<br>**4**<br>**5**<br>**6**	0<br>1 (4)<br>1 (4)<br>4 (17)<br>17 (71)<br>1 (4)	0<br>2 (6)<br>1 (3)<br>2 (6)<br>30 (86)<br>0	0.45
**Reacting pupils, n (%)**	13 (54)	26 (74)	0.16
**Traumatic SAH, n (%)**	22 (92)	28 (80)	0.29
**Epidural Hematoma, n (%)**	15 (63)	30 (86)	0.06
**Hypotension, n (%)**	6 (25)	16 (46)	0.17
**Hypoxemia, n (%)**	13 (54)	21 (60)	0.79
**Sodium, mmol/L**	140 (138-142)	137 (135-140)	0.04
**Glucose, mg/dL**	134 (116-177)	131 (122-167)	0.62
**Hemoglobin, g/dL**	12.2 (10.3-14.5)	12.2 (10.3-14.5)	0.88
**Mechanical ventilation, n (%)**	24 (100)	35 (100)	-
**Vasopressors, n (%)**	19 (79)	33 (94)	0.11
**Inotropes, n (%)**	3 (13)	6 (17)	0.73
**RRT, n (%)**	0	1 (3)	0.99
**EVD, n (%)**	24 (100)	27 (77)	0.02
**Intracranial hypertension, n (%)**	18 (75)	16 (46)	0.03
**Brain tissue hypoxia, n (%)**	NA	24 (69)	-
**Seizures, n (%)**	7 (29)	8 (23)	0.76
**TIL score basic**<br>**1**<br>**2**<br>**3**<br>**4**	6 (25)<br>9 (38)<br>4 (17)<br>5 (21)	9 (26)<br>7 (20)<br>6 (17)<br>13 (37)	0.42
**Osmotic therapy, n (%)**	14 (58)	18 (51)	0.79

**Hypothermia, n (%)**	7 (29)	8 (23)	0.76
**Barbituric, n (%)**	3 (13)	4 (11)	0.99
**Decompressive craniectomy, n (%)**	5 (21)	11 (31)	0.55
**ICU LOS, days**	6 (2-14)	16 (9-25)	0.002
**Hospital LOS, days**	12 (2-50)	30 (14-66)	0.03
**GCS at ICU discharge**	3 (3-12)	10 (3-14)	0.36
**ICU death, n (%)**	13 (54)	13 (37)	0.29
**Hospital death, n (%)**	13 (54)	13 (37)	0.29
**GOS at 3 months**	1 (1-3)	3 (1-4)	0.15

COPD = chronic obstructive pulmonary disease; APACHE: acute physiology and chronic health examination; SOFA: sequential organ failure assessment; GCS: Glasgow coma scale; GOS: Glasgow outcome scale; ICU: intensive care unit; LOS: length of stay; TIL: therapy intensity level; RRT: renal replacement therapy; EVD: external ventricular drainage; SAH : subarachnoid hemorrhage

Supplemental Figure S1: Panel A) Number of patients included in the study each year, according to the monitoring strategy (ICP or ICP/$PbtO_2$); Panel B) Percentage of non-survivors each year, according to the monitoring strategy (ICP or ICP/$PbtO_2$); Panel C) Percentage of patients with unfavorable neurological outcome at 3 months (Glasgow outcome scale 1-3) each year, according to the monitoring strategy (ICP or ICP/$PbtO_2$).

A)

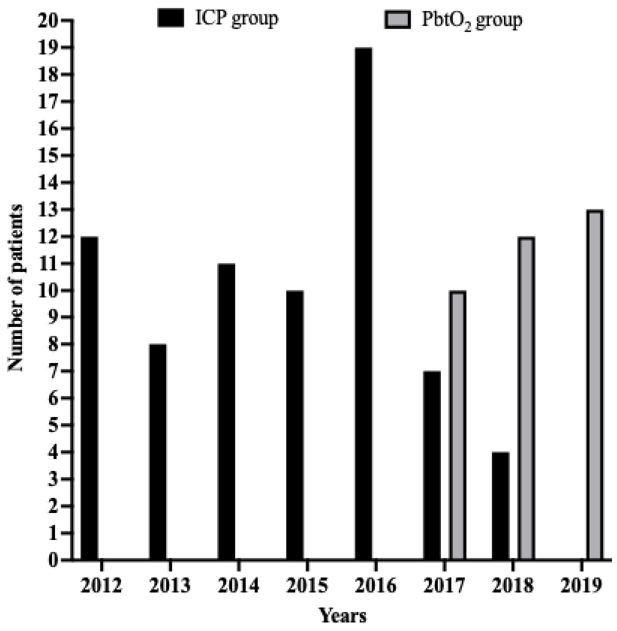

B)

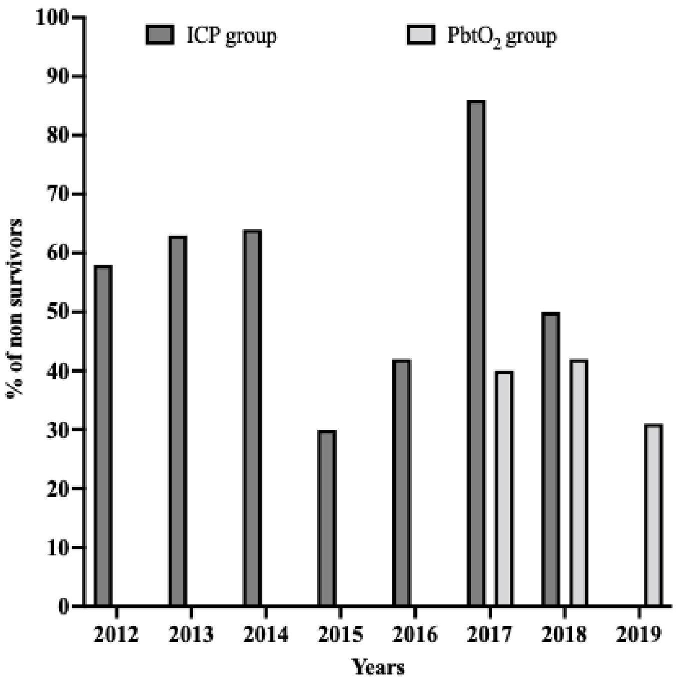

C)

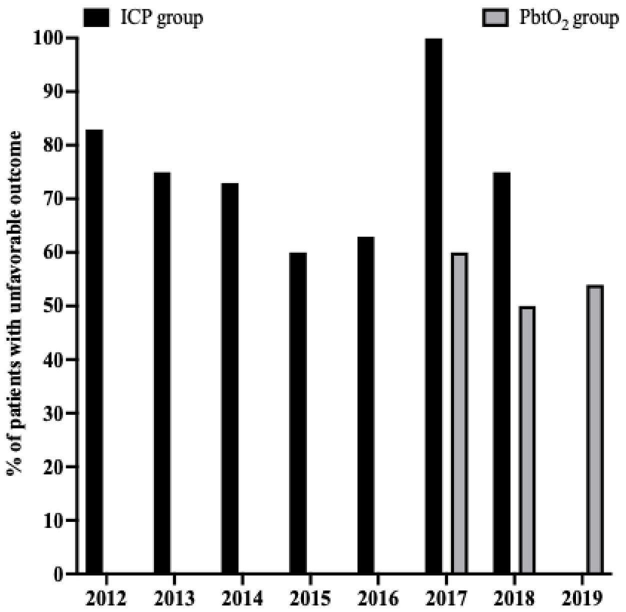
ICP group
PbtO2 group
% of patients with unfavorable outcome
100
90
80
70
60
50
40
30
20
10
0
2012
2013
2014
2015
2016
2017
2018
2019

Supplemental Figure S2: Kaplan Meier curve of survival in ICP and ICP/$PbtO_2$ group.

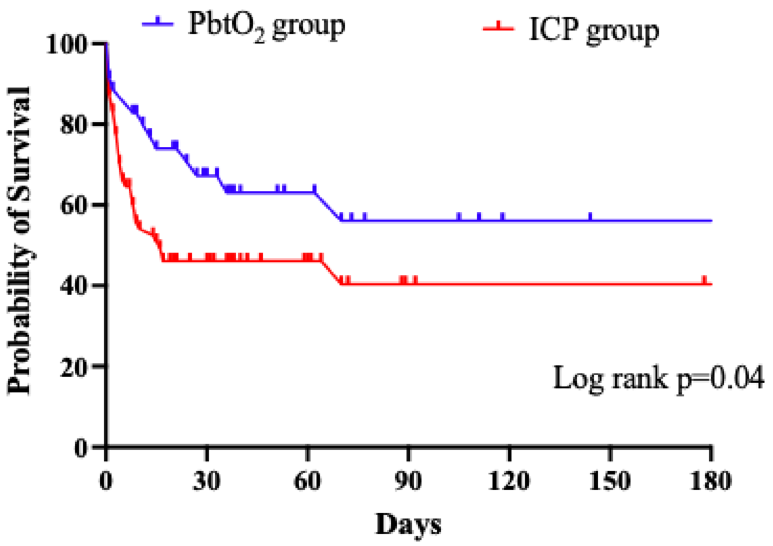

Supplement: Supplementary file 1 [file brainsci-12-00887-s001.zip › brainsci-1797588-supplementary.pdf]
